# An innovative method for clinical practice guideline contextualisation for chronic musculoskeletal pain in the South African context

**DOI:** 10.1186/s12874-019-0771-3

**Published:** 2019-06-28

**Authors:** D. V. Ernstzen, S. L. Hillier, Q. A. Louw

**Affiliations:** 1 Division of Physiotherapy, Faculty of Medicine and Health Sciences, Stellenbosch University, PO Box 241, Cape Town, 8000 South Africa; 2 Faculty of Medicine and Health Sciences, Stellenbosch University, P O Box 241, Cape Town, 8000 South Africa; 3 Division of Health Sciences, University of South Australia, Adelaide, 5000 Australia

**Keywords:** Clinical practice guideline, Contextualise, Musculoskeletal pain, Primary care

## Abstract

**Background:**

Clinical guidelines produced in developed nations may not be appropriate in resource-constrained environments, due to differences in cultural, societal, economic and policy contexts. The purpose of this article is to describe an innovative and resource-efficient method to develop a clinical practice guideline (CPG), using the CPG contextualisation approach.

**Methods:**

The four phased contextualisation framework was applied to produce a contextualised, multidisciplinary CPG for the primary health care of adults with chronic musculoskeletal pain (CMSP) in the South African context. The four phases were: a contextual analysis, evidence synthesis, contextual integration and external evaluation. Qualitative methodology was used to investigate context factors influencing health care in this environment. A systematic review was conducted to identify current, high-quality CPGs on the topic, and to synthesise a core set of clinical recommendations from the CPGs. Consensus methods were used to integrate context information with recommendations. A multidisciplinary panel of local experts authenticated and contextualised recommendations. The resultant CPG was externally reviewed using a survey.

**Results:**

The results from the contextual analysis phase indicated a wide range of contextual factors that could influence the applicability and implementability of the recommendations, including: the personal characteristics of the patient and clinician, social and environmental circumstances, healthcare interventions available, and healthcare system factors. During phase two, six existent high quality CPGs were identified and a core set of multidisciplinary recommendations were sourced from them. The contextual integration phase produced the validated recommendations, accompanied by its underpinning body of evidence and context specific information. The outcome of phase four (external review) was that the recommendations were confirmed as relevant for the intended setting.

**Conclusion:**

CPG contextualisation was found to be a practical approach to develop a contextualised multidisciplinary CPG for the primary health care of adults with CMSP in a South African setting. The contextualisation approach enhanced the integration of multiple stakeholder perspectives and highlighted the importance of considering clinical, social and economic complexities during CPG development. Attention to contextual information is advocated to enhance the uptake of CPG recommendations, particularly in resource constrained settings.

**Trial registration:**

Health Research Ethics Committee of Stellenbosch University, South Africa (S14/01/018); the review protocol was registered on PROSPERO (registration number CRD42015022098).

**Electronic supplementary material:**

The online version of this article (10.1186/s12874-019-0771-3) contains supplementary material, which is available to authorized users.

## Background

The implementation of clinical practice guidelines (CPGs) is advocated to optimise the quality, consistency, appropriateness and cost-effectiveness of health care [1]. CPGs are described as a set of systematically developed clinical recommendations that assist different stakeholders in making decisions about health care for specific clinical circumstances [2]. Clinical recommendations should be based on best available evidence to inform policy and practice by providing information regarding effectiveness, value and harms of interventions [3]. However, the uptake and effectiveness of CPGs are often questioned. Several studies have found mixed results regarding the ability of CPGs to influence practice patterns, patient outcomes and health system outcomes [4–7]. Various reasons are offered for the lack of CPG uptake. Kastner et al. [8] mention that implementation strategies for complex interventions, such as CPGs, have focussed on extrinsic factors to change provider behaviour and practice environments, rather than intrinsic guideline characteristics (e.g. clarity, specificity and clinical applicability of recommendations). Improving the applicability of the recommendations for the intended setting by integrating contextual factors during CPG development and implementation is suggested to enhance CPG uptake [7].

CPG contextualisation is a new and innovative approach to develop CPGs and to facilitate uptake in resource-constrained environments. Contextualisation is based on the premise that CPGs produced in developed nations may not be appropriate in resource-constrained environments, due to differences in the healthcare systems, socio-cultural, societal and policy contexts [9, 10]. There are several methods to develop CPGs: they can be written de novo, or existing guidelines can be modified [9–13]. In alternative CPG development, a guideline can simply be *adopted* to another application or environment; or they can be *adapted* (modified) in accordance with varying needs or circumstances. In the literature these processes of adoption and adaptation have primarily been performed in comparable, developed-nation contexts [9, 14]. A hybrid process has emerged from GRADE (Grading of Recommendations Assessment, Development and Evaluation) called Adolopment which combines elements of de novo, adoption and adaptation [12]. To deepen the ability to consider the often profoundly different circumstances of economics, service frameworks and demography of developing countries, the process of *contextualisation* was created [9]. During contextualisation, recommendations are sourced from existing CPGs, but are tailored to suit the specific needs of the context. A defining feature of the contextualisation process is that existing recommendations are not updated, but are rephrased to be suitable for the intended setting.

This study adds to the body of knowledge for CPG contextualisation by proposing a feasible way to explore and integrate context-related factors into the guideline development process. Context factors represent the typical circumstances into which interventions will be implemented, such as the setting in which people receive healthcare services [15]. Additionally, this study added the integration of multiple stakeholders’ perspectives, as several authors advocate the inclusion of stakeholder values and preferences as part of CPG development. However, the above strategy has not been commonly adopted in CPG development [16, 17]. Our contextualisation approach is different to previous contextualisation approaches [9], as we added a contextual analysis phase and we used consensus methods to endorse recommendations and integrate contextual information. For practical purposes, we explored the concept of CPG contextualisation using the example of chronic musculoskeletal pain (CMSP) in South Africa (SA), an upper-middle-income country with a transforming healthcare system [18, 19].

CMSP, a global healthcare concern, is a major cause of disability and morbidity in sub-Saharan Africa. In SA, musculoskeletal conditions contribute significantly to the years lived with disability (YLD) [20]. However, the current healthcare context of SA is challenged by the country’s quadruple burden of disease which consists of the prevention and management of the human immunodeficiency virus/acquired immune deficiency syndrome (HIV/AIDS) and tuberculosis; chronic non-communicable diseases; maternal and child health; and trauma and violence [19]. Consequently, healthcare resources are channelled towards these priorities, creating a resource scarcity to prevent and manage musculoskeletal conditions [21]. There is thus a need for evidence-informed, cost-effective and time-efficient management strategies to address CMSP and its consequences within the realities of the transforming SA primary healthcare sector.

Considering the need for the guideline and the array of context factors that may influence pain management in SA, the contextualising approach was proposed. A contextualised CPG was anticipated to offer a bridge between policy and best available evidence, whilst considering local circumstances, service provision factors, clinical expertise and patient choice [22, 23]. The purpose of this article is to describe a novel, practical and resource-efficient method to produce a CPG. The end product was a contextualised, evidence-informed, multidisciplinary CPG for the primary health care of adults with CMSP. We purposefully report on the objectives of our four-phased stepwise contextualisation process, summarised in Table 1. The objectives for each phase were:

- To develop a framework of contextual factors that influence the primary health care of patients with CMSP in a SA context.
- To identify existing, current, high-quality CPGs for the primary health care of CMSP and to synthesise core recommendations from these guidelines;
- To endorse and contextualise clinical recommendations for inclusion in the CPG; and
- To ascertain if the contextualised CPG is acceptable and feasible for the intended setting.

**Table 1:** Summary of different study phases for the contextualisation process

	Study phase 1	Study phase 2	Study phase 3	Study phase 4
Research design	Exploratory, descriptive, qualitative study	Systematic review	Consensus study	Small scale survey
Focus	The lived experience of patients with CMSP and the primary health care received. Healthcare practitioners’ perspectives about the primary health care of patients with CMSP.	Identification and appraisal of available CPGs and associated clinical recommendations for the primary health care of adults with CMSP.	Evaluation and endorsement of the clinical recommendations sourced during study phase 2. Development of context and practice points for implementation of the recommendations.	Stakeholders evaluated the applicability and acceptability of the draft CPG.
Setting	Three diverse community health centres/clinics in the public healthcare sector.	Guidelines specific to primary healthcare settings.	The local primary healthcare context.	The local primary healthcare context.
Sample eligibility	Adults with CMSP who presented for care at the indicated clinics. Healthcare practitioners involved in the management of adults with CMSP at the clinics.	CPGs on the topic that was available in full text and published during the timeframe January 2000 to May 2015.	Local healthcare experts who had practical experience and interest in CMSP. Practitioners from diverse settings (government health subdivisions, academic institutions and private practitioners).	A group of potential end-users such as policy makers, representatives from professional organisations and clinicians.
Procedures/Instrumentation	Semi-structured individual interviews. Participants completed a sociodemographic questionnaire. Patient participants completed a questionnaire on pain location and intensity, the pain disability index and the Kessler psychological distress scale.	Systematic search and selection. Quality appraisal using the AGREE II instrument. Data extraction into a recommendations matrix.	Online Delphi survey in two rounds, interspaced with a consensus meeting. Delphi surveys: the panel evaluated and rated each recommendation for its applicability for the SA context. Consensus meeting: the panel members worked in focused groups to generate context points, using the information obtained from study phase 1.	Participants appraised a short form of the draft CPG using a questionnaire. Feedback on the following were invited: The endorsed recommendations; context points; the proposed patient pathway; acceptability for patients and staff, healthcare resources, training required and format.
Data analysis	Inductive, thematic, content analysis. The questionnaire data were analysed and conveyed as frequencies, proportions and percentages.	Methodological quality: Summary of domain scores obtained for AGREE II using the principles provided in the user manual. Content analysis comprising of: recommendation content, wording, underpinning body of evidence and references.	Delphi survey: explicit aggregation using the median as a measure of central tendency and the interquartile range (IQR) for the level of dispersion. Consensus meeting: documented context points were categorised and thematically summarised.	Ordinal data were summarised using the median and IQR, while interval data were summarised using the mean and standard deviation.
Main findings	Framework of contextual factors that influence pain management in this context. Barriers to and facilitators of pain management.	A set of multidisciplinary clinical recommendations as propositions for inclusion in the CPG.	A core set of recommendations were endorsed by the panel. Context points for implementation of the recommendations.	Confirmation of applicability and acceptability of recommendations in the intended context. Identification of key topics that need further exploration.
Use of information in the study	Used in study phase 3 to inform decision making and to develop context points.	The clinical recommendations formed the foundation of study phases 3 and 4.	The endorsed recommendations were included in the draft guideline and used during study phase 4.	Refining the CPG.

*AGREE II* Appraisal of Guidelines Research and Evaluation version II, *CPG* Clinical Practice Guideline, *IQR* Interquartile Range, *CMSP* Chronic Musculoskeletal Pain

## Methods

### Study design

**Fig. 1:**
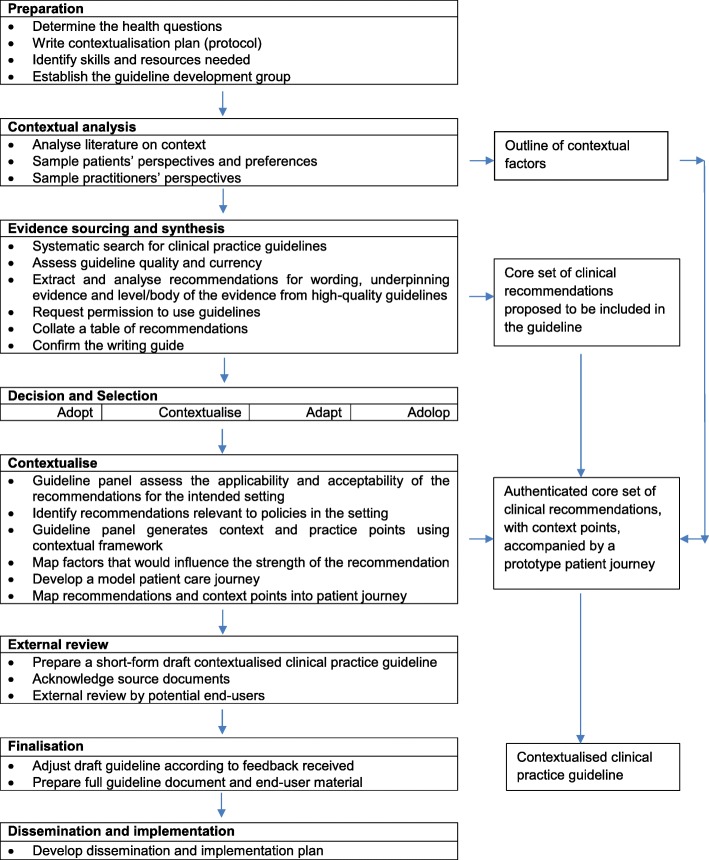
Stepwise process followed during the contextualisation process

We performed a four phased CPG contextualisation process, each phase using different research methods (see Fig. 1 and Table 1), drawing on the principles of knowledge translation research. Knowledge translation has been described as an iterative approach for improving healthcare delivery, utilisation and outcomes. This iterative process involves synthesising relevant research, interacting with users to identify needs and barriers for implementation, employing tailored strategies to promote adoption of evidence-based recommendations, and evaluating or monitoring their impact [24, 25]. Based on the principles of knowledge translation, the contextualisation process was designed to include stakeholder interaction through a contextual analysis (phase 1), sourcing and synthesis of evidence (phase 2), contextual integration to validate recommendations and indicate factors that would influence implementation of recommendations (phase 3) and an external evaluation of the proposed CPG (phase 4). A comprehensive description of the methods and findings is available in Ernstzen [26]; and a glossary of terms used in this manuscript is available in Additional file 1.

#### Phase 1: contextual analysis

We conducted two qualitative descriptive studies, one focusing on patients’ perspectives of CMSP and the primary health care they received; and the other focussing on healthcare practitioners’ perspectives on the management of CMSP in primary healthcare. The aim was to explore contextual factors that influence pain management in this setting. The study setting was the Western Cape, one of the nine provinces in SA. Three community health centres/clinics in the public healthcare sector were strategically chosen as study sites, based on their geographical location. The three clinics represented a rural, a semi-urban and a township setting, which allowed for diversity. This phase created a participatory opportunity for patients and clinicians in the contextualisation process [16].

#### Phase 2: evidence sourcing and synthesis

Phase 2 focused on the identification and synthesis of strategies for the management of CMSP [3, 10]. A systematic review was conducted to identify and appraise existing, up-to-date CPGs for the primary health care of CMSP. Ernstzen et al. [27] provides a complete report of the systematic review process and findings. Our priori questions were framed using the PIPOH (Population, Intervention, Professions, Health Outcomes and Health setting) format for guideline reviews [10]. The questions were therefore related to any intervention or strategy that were used to evaluate, diagnose and manage CMSP and its consequences in the primary health care setting. The outcomes of interest could include patient outcomes, system outcomes or public health outcomes. We did not have priori clinical questions, but rather created the content based on the clinical recommendations contained within the existing CPGs. We searched 13 guideline clearinghouses and five online databases using predetermined keywords. The methodological quality of CPGs that complied with the inclusion criteria was assessed using the AGREE II (Appraisal of Guidelines Research and Evaluation, Version II) [28]. The AGREE Enterprise [28] does not provide cut-off scores to differentiate between high-quality and low-quality CPGs. They advise that the decision about cut-off scores for CPG quality should be made by the user and taking the context into account. We analysed the content of CPGs that achieved a median score of 50% or more for the AGREE II domain regarding rigour of development. The cut-off was applied to ensure credibility of content in the contextualised CPG.

Clinical recommendations were extracted from the high-quality CPGs into a recommendations matrix for analysis. We extracted the recommendation, its assigned level of evidence, the strength of the recommendation and references supporting the recommendation [9, 10]. Recommendations were evaluated and synthesised according to the key principles for contextualisation [9, 14]. The consistency of content of recommendations, the level of evidence for each recommendation, the volume and currency of underpinning evidence and the strength of the recommendation were evaluated. The synthesis process involved categorising and organising similar recommendations extracted from different CPGs together; followed by merging the wording, evidence levels, and combining evidence sources to form one composite recommendation. Care was taken not to change the meaning of a recommendation when reformulating. A writing guide was used to be explicit about combining and communicating the levels of evidence (Table 2) [9, 14, 29]. The ‘levels of evidence’ is a hierarchical system that classifies evidence according to different study designs [30]. The synthesis process was performed to consolidate the variety of recommendations into a core set of recommendations for inclusion in the CPG.

**Table 2:** Writing guide to phrase endorsements (adapted from the Philippine Academy of Rehabilitation Medicine [14]; Gonzalez-Suarez et al. [9], with permission)

Phrase for strength of evidence	Description of type level of evidence	Guide for writing endorsements
There is strong evidence	Consistent grades of high level of evidence with uniform thought,^a^ and at least a moderate volume of references to support the recommendation.	We strongly recommend
There is evidence	A mix of moderate- and high levels of evidence with uniform thought and at least a low volume of references. A mix of high- and low - levels of evidence with uniform thought and high volume of references. High level of evidence coupled with good practice points (GPPs), and at least moderate volume of references. Consistent grades of high level of evidence with uniform thought, and at least a low volume of references. One high level of evidence study (systematic review) and at least a moderate volume of references.	We recommend
There is some evidence	One moderate level of evidence study (Randomised controlled trial). Inconsistent high and low levels of evidence with uniform thought and a moderate volume of references. Inconsistent moderate and low levels of evidence with uniform thought and a moderate volume of references. Consistent grades of moderate levels of evidence and GPP with uniform thought and at least a moderate volume of references. Consistent grades of low levels of evidence with uniform thought and at least a moderate volume of references.	
There is conflicting evidence	Mixed levels of evidence with non-uniform thought, irrespective of the volume of references.	We suggest that clinicians consider^b^
There is limited evidence	A mix of levels of evidence with uniform thought, irrespective of the volume of references with or without GPPs. Consistent grades of moderate levels of evidence with uniform thought and a low volume of references.	
There is expert consensus that it is good practice	GPP only (no evidence): based on expert consensus.	
There is insufficient/no evidence	Low or mixed levels of evidence with a low volume of references with or without GPPs. Absence of evidence.	We do not endorse

*(GPP = General practice point)*

^a^Where only one recommendation is present, the criterion of uniformity of thought cannot be adhered to and therefore does not apply

^b^In the absence of a strong evidence base, but where plausible hypotheses exist for a particular recommendation (such as theoretical explanations, physiological rationale, expert consensus or other forms of such data), the clinician should use his/her own discretion by applying clinical reasoning to make a decision

#### Phase 3: contextual integration

Consensus methodology, consisting of a modified Delphi approach, was used for the contextual integration phase [3, 31–33]. The information derived from the contextual analysis and the evidence synthesis were used in phase 3. A multidisciplinary panel of experts were invited to evaluate and validate the proposed recommendations, considering the applicability and acceptability of the recommendations for the SA setting. An online Delphi survey with two rounds was implemented, interspaced with a consensus meeting. The results from the first online survey were discussed during the consensus meeting before entering the second Delphi round. Additionally, during the second half of the consensus meeting, the panel members worked in focus groups to generate and document context points for each recommendation, using the format for contextualisation framed by Gonzalez-Suarez et al. (2012) [9] (Table 3). The endorsed recommendations with their context points were organised into an authentic patient pathway. Stakeholder feedback from phase 1, regarding optimisation of the patient journey through the healthcare system, were considered in developing the patient pathway. The panel members were requested to declare any actual or potential conflicts of interest that may have had a direct influence on the content of the recommendations. Any financial, professional affiliation and/or intellectual conflicts of interest had to be stated to identify any potential sources of bias.

**Table 3:** Example – Context and Practice points for recommendations on advice and education

TOPIC	Strength of the evidence	Recommendation Endorsement Statements for ADVICE AND EDUCATE
Address concerns	There is evidence	*We recommend* that clinicians address the patient’s concerns; and beliefs and teach the person, their family and caregivers about pain management strategies. ^a^ *Involve the family in education to enhance support. Provide relevant patient education material.*
Brief education	There is evidence	*We recommend* that brief education be given to patients with chronic musculoskeletal pain to facilitate continuation of work/occupation. ^a^ *Brief education can be on: examination, information, reassurance and advice to stay active.*
Advice to stay active	There is evidence	*We recommend* advice to stay active in addition to exercise therapy for patients with chronic low back pain to minimise long-term disability. ^a^ *Encourage occupational activities where indicated.*
Therapeutic neuroscience education	There is expert consensus^b^	*We suggest that the clinician consider* pain neuroscience education to assist the patient in understanding their condition, change their conception about pain and improve their ability to cope with pain [34]^a^. ^a^ *Use narratives and language that are applicable to the local context and that are culturally appropriate.*
Education about analgesia	There is evidence	*We recommend* that the clinician: - educate patients about the risks and benefits of all medications and - monitor and manage side-effects. ^a^ *Use educational material for patients. Consider advice about concomitant use of over-the-counter medicines and herbal remedies.*
Source guidelines		Institute for Clinical Systems Improvement (ICSI) [35]; National Opioid Use Guideline Group (NOUGG) [36]; Scottish Intercollegiate Guidelines Network (SIGN) [37].
Criterion		Context and practice points
Organisational		Early education is important. Educational component can be delivered as part of a group intervention. Access to work sector to deliver educational strategies and material on occupational health is needed.
Practice method (how)		Verbal or written clear instructions; specific to condition Promotive: educational sessions at the worksite; need formal work assessment The educational interventions should be culturally appropriate.
Staff (who)		All treating clinicians can provide educational interventions. Work interventions require more attention/focus.
Resources		Printed educational material for patients should be available. Refer to trustworthy e-sources. Audio-visual material such as educational videos in waiting areas can be useful.
Training		The following training opportunities should be provided to enhance educational interventions: motivational interviewing skills, communication skills training, basic health promotion training. Occupational health training where needed; vocational training. May need training in pain neuroscience education.
Timing (when)		Needs to be given from an early stage of the management programme. Advise and educate at first consultation, but can be a continuous process.
Re-assessment		Assessment of recall and adherence to advice and education should take place as part of usual care at each session as appropriate. Vocational assessment should be done where indicated.
Referral		Within the interdisciplinary team
Patient/family		Explain findings of assessment to the patient using appropriate language. Patient education is important to foster adherence to treatment. Family education may enhance support. Educate patient and family about benefits of staying active and about pain neuroscience. Educate employers and colleagues at the workplace.
Policy		Healthcare 2030 [38] supports a patient-centred approach

^a^ *Practice points*

^b^ *Recommendation nominated by the expert panel*

#### Phase 4: external review

An external review via a small-scale survey was done to obtain the views of different stakeholders about the acceptability and applicability of the CPG for the intended setting [9, 11, 39, 40]. A diverse group of potential end-users, consisting of representatives from government departments, professional organisations and clinicians from a primary healthcare centre, were invited to review the draft version of the CPG. The list of endorsed recommendations, together with their level of evidence, context points and accompanying patient pathway, were presented to the reviewers, along with a questionnaire. The questionnaire contained questions about the applicability and acceptability of the CPG [12]. Criteria for applicability comprised organisational context, availability of health services and expertise, population characteristics, beliefs, and values. Questions regarding acceptability focused on strengths and weaknesses of the CPG, suggestions for modification, impact on current routines, training required, barriers and facilitators, resource implication, and practicality. Patients were not included in the external review; as patient consultations were envisaged to be part of an implementation plan and development of end-user documents.

## Results

### Phase 1: contextual analysis

Twenty patients with CMSP and 21 clinicians participated in the interviews for the contextual analysis. The findings indicated that CMSP influenced patients in multiple ways. Participants largely agreed on the range of context factors that influence CMSP care. Components of the contextual framework which were reported to impact on the treatment delivery and adherence to care included: patients’ beliefs, goals, expectations, coping strategies, needs regarding empowerment and preparation for self-management, family support, occupational influences and financial considerations. Additionally, service provision and the application of best available evidence was influenced by practitioner beliefs, training, availability of resources, staff shortages and turnover, access to care and healthcare system load. These context factors presented important information that were utilised in the contextualisation process and they are potentially useful for designing an implementation plan for the CPG.

### Phase 2: evidence sourcing and synthesis

Twelve clinical guidelines on the primary health care of CMSP were identified through the systematic review [27]. Six of these clinical guidelines were of high quality, and 156 recommendations were extracted from them. The end-result after the synthesis process was a core set of 43 multidisciplinary, clinical recommendations. The main reasons for the reduction in the number of recommendations were merging of similar recommendations (to limit repetition) and exclusion of recommendations that were not available or relevant in the SA primary healthcare context. The latter consisted mainly of opioid prescriptions. The content of the core set included recommendations on: approach to care, assessment, educational interventions, referral, pharmacological management, physical therapy, electrotherapy, psychological therapy, complementary therapy, and self-management. Eight out of the 12 (67%) CPGs included indications for the level of the evidence underpinning recommendations, while five (42%) provided a grading system for rating the strength of the recommendation.

### Phase 3: contextual integration

Seventeen panel members from a range of professions participated in round one of the Delphi process to evaluate the recommendations. Fourteen members participated in round two, while 13 participated in the consensus meeting. The panel of local experts comprised of clinicians, academics and researchers. The professions represented were physiotherapy, nursing, medicine (family medicine, anaesthesiology), occupational therapy, psychology, and medical anthropology. The panel of experts, although from diverse backgrounds, professions and work sectors, reached consensus on 42 statements. The panel rated one statement as undecided. Two recommendations were not validated due to limited evidence for efficacy; unclear benefit vs harm relationship and context factors such as cost and availability of resources. The panel nominated an additional recommendation for inclusion, which was included in Delphi round 2, based on emerging evidence for that recommendation. It was noted that there is a need for additional recommendations, which were not included in the source guidelines, but were deemed applicable to the SA context. Examples of such recommendations include: socio-environmental strategies, management of multi-morbidities and team organisation.

During the consensus meeting, the panel generated context and practice points for the implementation of each CPG recommendation in the intended setting. The context points represented the facilitators, barriers, requirements and possible solutions for implementation of the recommendations. These context criteria summarised how pain care can be optimised and were comprised of features of organisation of care, interdisciplinary referral and communication, processes of care skill and training required, access to care, local available resources, equipment required, patient and family involvement and policy factors (Table 3). Work based interventions, the accessibility of rehabilitation services and culturally appropriate tools for interventions were prominent context factors to be considered when implementing the recommendations.

### Phase 4: external evaluation

The 18 external reviewers, representing a variety of clinicians and four professional organisations, confirmed the content of the CPG to be largely applicable and acceptable for the intended context. The reviewers highlighted similar barriers to, and facilitators for implementation as identified in Phase 3. Training of primary care providers to use the CPG and to implement its recommendations was listed as a key prerequisite for ensuring successful implementation and achieving important outcomes.

## Discussion

The study adds to the body of knowledge of CPG contextualisation as an innovative methodology in the field of CPG development [9]. We investigated a method to contextualise CPGs that were developed in a variety of settings, for use in the SA setting. Contextualisation was indicated considering the difference in contexts of the existing guidelines developed in high-income countries with well-developed healthcare systems, for application in SA, an upper-middle-income country with a transforming healthcare system [19].

A strength of the contextualisation process was the rigorous approach used to gain information about the context of the intended setting. Context plays a role in health behaviour change, knowledge translation, development of context-specific interventions, implementation of interventions and health outcomes [7, 12, 13]. Contextual knowledge considers the real world (authentic) circumstances within which the CPG will be implemented [7, 12, 15] and is therefore an essential component to consider during CPG development. However, there is a shortage of contextual information about specific healthcare environments that would be useful for CPG adoption and adaptation [12, 13]. Sav et al. [41] argues that there is scant evidence for contextual factors in developing countries and culturally different populations. The contextualisation approach accounted for this lack of information by including a systematic situational analysis; however, more research on context factors that influence health care provision is required to diversify the knowledge base. The context information generated in our study was used to incorporate clinical recommendations in a locally applicable clinical pathway and to design context and practice points which framed the recommendations to be relevant to the local context.

Stakeholder perspectives are a key consideration when developing contextually relevant CPGs [22]. Recent studies on alternative guideline development did not focus on including patient perspectives [42–44]; with some authors citing cost and organisational factors as limiting factors. Schünemann et al. 2017 [12] included patient representatives for their adolopment process. We used a different approach for including patients as stakeholders, by investigating patients’ perspectives on current care and their preferences and needs for optimised pain care. Our findings confirmed the notion that the consideration of patient perspectives in CPG development may enhance patient-centredness and culturally relevant considerations [13, 16, 29]. Congruently, involving clinicians was found to be useful to identify potential training needs and barriers and facilitators regarding uptake of the recommendations. Healthcare practitioners were included in multiple ways in our contextualisation approach, via the contextual analysis, the contextual integration and review phases. During the contextual integration phase, the practitioners used their background knowledge about policies, regulatory requirements, healthcare system factors, workforce considerations and resources, processes of care, feasibility and optimal practice in their decision making [12, 13]. This contextual integration was important, since Gandhi et al. [45], found that the recommendations contained in CPGs they reviewed, were not sensitive to the resource limitations and context factors of low- and middle-income countries (LMIC). Explicitly stating contextual information is not reported to be a key feature of similar alternative CPG endeavours [12, 14, 42–44]; and the inclusion there-of is a recommendation for future practice. Providing context information and creating an authentic clinical pathway, may facilitate the uptake of CPG recommendations, clinical decision making and quality of health care [33].

Systematically reviewing existing CPGs to produce an evidence synthesis, is central feature of alternative CPG development [9–12]. In our case, synthesising CPG recommendations from different source CPGs lead to the formation of a hybrid set of multidisciplinary recommendations, which enhanced the holistic scope of the CPG [27]. This feature is imperative, considering the multidimensional impact of CMSP on wellbeing [46]. Assimilation of recommendations from an assortment of high-quality CPGs has been used before by Zhang et al. [3], to develop a core set of recommendations for the management of hip and knee osteoarthritis. A disadvantage of the approach is that the recommendations contained in the contextualised CPG are limited to those contained in the source CPGs. While the expert panel could nominate prioritised additional recommendations, developing new recommendations was beyond the scope of our contextualisation approach. The merging of the recommendations was uncomplicated in our study, however, the assimilation of the body of evidence presented a challenge due to the inconsistent use of frameworks to aggregate the body of evidence and the strength of the recommendation [27]. We therefore formulated context and practice points to indicate elements that may influence the strength of the recommendation. The strength of the recommendation is influenced by the certainty of evidence, and by contextual factors such as acceptability, feasibility, importance of outcomes and resource implications [12]. We suggest a formal process to grade the strength of the recommendation for future contextualisation endeavours as used by similar studies, for example: the GRADE evidence to decision (EtD) framework [12]; the FORM framework (Australian method for formulating and grading recommendations) [30]; or a visual analogue scale rating method [3]. The use of a frameworks and a writing guide is fundamental to ensuring consistency in the CPG development.

### Strengths and limitations

This study highlights the strengths and the limitations of the contextualisation approach. Contextualisation has been advocated as a method to use available resources more efficiently, thereby permitting resources to be used for implementation [9, 10]. Time and resources were saved since the systematic review focused on identifying existent, high-quality CPGs, eliminating the process of systematic reviews for different healthcare questions required for a de novo CPG. The process was driven by a core team, with multiple stakeholder input, which, together with the use of electronic voting, negated the need for multiple group meetings. Future research should focus on the extent to which contextualisation contribute to the implementation and uptake of CPGs, since context-specific factors has been considered during the development process.

We outlined the steps we followed to contextualise CPG recommendations for the primary health care of patients with CMSP (Fig. 1, Table 1). However, we acknowledge that CPG contextualisation, by nature, is a multifactorial and context specific process. Reproducing the process we followed may not be indicated or feasible in a particular context or for a specific health condition. We endeavoured to provide generic principles that can guide a contextualisation process; however, variations may be required to enhance the development of relevant CPGs and their implementation in LMICs. The overarching contextualisation approach is flexible, to create a CPG development process that is fit for purpose; yet adheres to guiding methodological principles. Examples of the fit for purpose approach is evident in the alternative guideline development processes that has been used before [9, 42–44]. It is therefore important that a CPG contextualisation process should be relevant to the healthcare setting, clinical context and health condition.

The success of CPG contextualisation is dependent on the availability, quality, scope, and currency of the parent CPGs. If no up-to-date, high-quality, holistic CPG on the topic exists, the contextualisation process cannot take place, and de novo development is indicated. We took note of the authorisation information in some source guidelines, and contacted developers to request permission to use CPGs as part of the contextualisation process. However, few responded. Due to the relative novelty of CPG contextualisation, there is a need for communication with recognised CPG development bodies about the process and purpose of contextualisation. McGowan et al. [43] highlighted a similar concern regarding consent to adapt CPGs. The process of contextualisation requires methodological skills regarding obtaining and appraising CPGs, synthesising recommendations, contextual integration, and the facilitation of a consensus group. If access to these skills are not available, alternative options may have to be sought. The decision to de novo develop, adopt, adapt, contextualise, or adolop CPGs depend on various factors such as local cultural and organisational context, skills, timelines and resources available [9, 12]. Whichever option for CPG development is indicated, it remains important to adhere to the quality indicators for CPG development [40] and to consider contextual topics [7].

### Implications

The distinctive features of our four phased CPG contextualisation process were the emphasis on identification, integration and explicit statement of contextual factors that could influence the phrasing, implementation and uptake of CPG recommendations. We advocate the systematic integration of context information and multiple stakeholder input during CPG development to enhance the acceptability and implementability of CPGs in resource-constrained environments.

## Conclusion

CPG contextualisation was found to be a practical, logical, and time- and resource-efficient approach towards developing a contextualised evidence-informed, multidisciplinary guideline for the primary health care of adults with CMSP in a SA setting. The approach provided the opportunity to blend clinical recommendations and multiple stakeholder perspectives within the SA context. The advantages of the contextualisation method are the explicit statements about contextual factors and the identification of indicators for the successful implementation of the guideline. An area for development for the process comprises the formalisation of the use of the strength of the recommendation.

## Additional file

Additional file 1: A glossary to explain the concepts used in the study. (DOCX 19 kb)

## Data Availability

The full dataset is available from the author and can be accessed through the Stellenbosch University library (Ernstzen, 2017) [26].

## Abbreviations

AGREE II: Appraisal of Guidelines Research and Evaluation, Version II
CMSP: Chronic Musculoskeletal Pain
CPG: Clinical Practice Guideline
EtD: Evidence to Decision
FORM: An Australian method for formulating and grading recommendations in evidence-based clinical guidelines
GPP(s): Good Practice Point(s)
GRADE: Grading of Recommendations Assessment, Development and Evaluation
ICSI: Institute for Clinical Systems Improvement
LMIC: Low- and Middle-Income Countries
NOUGG: National Opioid Use Guideline Group
PARM: Philippine Academy of Rehabilitation Medicine
SA: South Africa(n)
SIGN: Scottish Intercollegiate Guidelines
