# Supplementary material for: An innovative method for clinical practice guideline contextualisation for chronic musculoskeletal pain in the South African context
Source: BMC Med Res Methodol. 2019 Jun 28;19:134. doi: 10.1186/s12874-019-0771-3 (PMC6599395; doi:10.1186/s12874-019-0771-3)
Supplement: Supplementary file 1 — A glossary to explain the concepts used in the study. (DOCX 19 kb) [file 12874_2019_771_MOESM1_ESM.docx]

**Glossary for:**

# **An innovative method for clinical practice guideline contextualisation for chronic musculoskeletal pain in the South African context**

**Adaptation of a guideline:** The use and/or modification of guideline(s) produced in one cultural or organisational setting for application in a different context. Adaptation can be used as an alternative to *de novo* guideline development or for customising existing guidelines(s) to suit the local context [1].

**Adolopment:** A combined approach for adoption, adaptation, and de novo guideline development based on the GRADE EtD frameworks [2].

**Adoption of a guideline:** The acceptance of a guideline as a whole after the assessment of its quality, currency and content. Healthcare providers (or other users of recommendations) who adopt a guideline, are committed to changing their practices in accordance with the recommendation in the guideline [1].

**Body of evidence/Quality of evidence:** The quality of evidence reflects the extent to which confidence in an estimate of the effect is adequate to support recommendations [3]. The evidence base (i.e. number, level and risk of bias in included studies, which forms the body of evidence) [4].

**Consensus development:** Formal consensus development methods are ways of obtaining and synthesising views of experts, opinion leaders and stakeholders. They involve the generation of group judgements based on explicit aggregation on individual participants’ judgements [5].

**Contextual factors:** Contextual factors include personal and environmental features of the individual. Personal factors are individualistic features such as gender, age, coping styles, social background, education, profession, past and current experience, overall behaviour pattern, and character, amongst other factors. The environmental factors comprise the physical, social, legal, external and attitudinal environment in which people live and conduct their lives. Contextual factors can act as facilitators of health care or barriers to such care [6].

**Contextualisation of a guideline:** Contextualisation of a guideline for use in developing countries means retaining its current form, and using writing strategies that assist in its operationalisation in the local environment. The focus is on how to best translate existing evidence statements into local practice [7].

**Evidence-based practice:** The conscientious explicit and judicious use of current best evidence in making decisions about the care of individual patients; the integration of best research evidence with clinical expertise and patient values [8].

**Knowledge translation research:** The scientific study of the determinants of knowledge use and the methods to promote the uptake of research findings by healthcare providers, policy makers and patients [9]. It is also known as implementation research and quality improvement research.

**Level of evidence:** A hierarchical system that classifies evidence according to different individual study designs [4].

**Primary health care:** A holistic approach to health care focussing on optimal health, well-being and equitable health care. PHC is focussed on individuals, family and community health care needs across the spectrum of health promotion, prevention, rehabilitation, curative and palliative services [10].

**Recommendations:** Evidence-based statements that promote or advocate a particular course of action in clinical practice [11].

**Rehabilitation**: A goal-orientated and time-limited process aimed at enabling impaired persons to reach an optimum mental, physical or social functional level [12].

**Strength of the recommendation:** The extent to which one can be confident that the desirable effects of an intervention outweigh the undesirable effects [13].

**REFERENCES:**

1. ADAPTE II Collaboration. 2009 (Updated 2010). *ADAPTE Resource Toolkit for Guideline Adaptation Version 2.0.* [Online] Available at: <http://www.g-i-n.net/document-store/working-groups-documents/adaptation/adapte-resource-toolkit-guideline-adaptation-2-0.pdf/view?searchterm=ADAPTE>. [Accessed 24 January 2019].
2. Schünemann HJ, Wiercioch W, Brozek J, Etxeandia-Ikobaltzeta I, Mustafa RA, Manja V, Brignardello-Petersen R, Neumann I, Falavigna M, AlHazzani W, Santesso N, Zhang Y, Meerpohl JJ, Morgan RL, Rochwerg B, Darzi A, Rojas MX, Carrasco-Labra A, Adi Y, AlRayees Z, Riva J, Bollig C, Moore A, Yepes-Nuñez JJ, Cuello C, Waziry R, Akl EA. GRADE Evidence to Decision Frameworks for adoption, adaptation and de novo development of trustworthy recommendations: GRADE-ADOLOPMENT. J Clin Epidemiol. 2017;81:101–110 . <http://dx.doi.org/10.1016/j.jclinepi.2016.09.009>.
3. Guyatt, G.H., Oxman, A.D., Vist, G.E., Kunz, R., Falck-Ytter, Y. & Schünemann, H.J. 2008. GRADE: what is “quality of evidence” and why is it important to clinicians? *British Medical Journal*, Volume 336: 995-8.
4. Hillier S, Grimmer-Somers K, Merlin T, Middleton P, Salisbury J, Tooher R, Weston A. FORM: An Australian method for grading recommendations in evidence-based clinical guidelines. BMC Med Res Methodol*.* 2011;11:23. <http://www.biomedcentral.com/1471-2288/11/23>.
5. Halcomb E, Davidson P, Hardaker L. 2008. Using the consensus development conference method in healthcare research. Nurs Res, 16(1): 56–70.
6. World Health Organization (WHO). 2013. *How to use the ICF: A practical manual for using the International Classification of Functioning, Disability and Health (ICF). Exposure draft for comment.* Geneva: World Health Organization.
7. Gonzalez-Suarez C, Grimmer-Somers K, Dizon J, King E, Lorenzo S, Valdecanas C, Gambito, E, Fidel B. Contextualizing Western guidelines for stroke and low back pain to a developing country (Philippines): an innovative approach to putting evidence into practice efficiently. J Healthc Leadersh*.* 2012;4:141–56.
8. Sackett DL, Rosenberg WM, Gray JA, Haynes RB, Richardson WS. Evidence based medicine: what it is and what it isn't. BMJ. 1996;312:71–2.
9. Eccles, M., Grimshaw, J., Walker, A., Johnston, M. & Pitts, N. 2005. Changing the behaviour of healthcare professionals: the use of theory in promoting the uptake of research findings. *Journal of Clinical Epidemiology*, Volume 58: 107–112.
10. World Health Organization (WHO). A vision for primary health care in the 21st century: towards universal health coverage and the Sustainable Development Goals. Geneva: World Health Organization and the United Nations Children’s Fund (UNICEF), 2018.
11. Misso, M.L., Pitt, V.J., Jones, K.M., Barnes, H.N., Piterman, L. & Green, S.E. 2008. Quality and consistency of CPG for diagnosis and management of osteoarthritis of the hip and knee: A descriptive overview of published guidelines. *The Medical Journal of Australia*, October, Volume 189: 394–399.
12. National Department of Health. 2015. *Framework and Strategy for Disability and Rehabilitation Service in SA (2015-2020)*, South Africa: National Department of Health.
13. Guyatt, G.H., Oxman, A.D., Kunz. R., Falck-Ytter, Y., Vist, G.E., Liberati, A. & Schünemann, H.J. 2008a. GRADE: going from evidence to recommendations. *British Medical Journal,* Volume 336: 1049 – 1051.
